# Vasorelaxing and antihypertensive activities of synthesized peptides derived from computer-aided simulation of pepsin hydrolysis of yam dioscorin

**DOI:** 10.1186/s40529-014-0049-3

**Published:** 2014-06-07

**Authors:** Yin-Shiou Lin, Yeh-Lin Lu, Guei-Jane Wang, Hong-Jen Liang, Wen-Chi Hou

**Affiliations:** 1 Graduate Institute of Pharmacognosy, Taipei Medical University, Taipei, Taiwan; 2 School of Pharmacy, Taipei Medical University, Taipei, Taiwan; 3 Graduate Institute of Clinical Medical Science, China Medical University, Taichung, Taiwan; 4 Department of Medical Research, China Medical University Hospital, Taichung, Taiwan; 5 Department of Health and Nutrition Biotechnology, Asia University, Taichung, Taiwan; 6 Department of Food Science, Yuanpei University, Hsinchu, Taiwan; 7 Traditional Herbal Medicine Research Center, Taipei Medical University Hospital, Taipei, Taiwan

**Keywords:** Angiotensin converting enzyme, Antihypertensive activity, Blood pressure, Vasorelaxing, Yam dioscorin

## Abstract

**Background:**

We reported that yam dioscorin and its peptic hydrolysates exhibited ACE inhibition and antihypertensive effects on SHRs, however, the active peptides are not really isolated until now. Using ACE inhibitory screenings, two penta-peptides, KTCGY and KRIHF, were selected for *ex vivo* and *in vivo* experiments.

**Results:**

KTCGY, KRIHF, and captopril were shown to have similar vasodilating effects against phenylephrine (PE)-induced tensions in rat endothelium-dependent thoracic aortic rings, however, KTCGYKTCGY (two-repeated KTCGY) and TCGYTCGY (two-repeated TCGY) were showed endothelium-independent vasodilating effects against PE-induced tensions. KTCGY, KRIHF (10 or 20 mg/kg), and captopril (10 mg/kg) were used to evaluate antihypertensive activity during 24-h after a single oral administration to spontaneously hypertensive rats (SHRs). The KTCGY and KRIHF showed significantly different and reduced the systolic blood pressure of SHRs compared to the blank.

**Conclusions:**

These results suggest that KTCGY and KRIHF may contribute important roles in yam dioscorin for regulating blood pressure *in vivo*.

**Electronic supplementary material:**

The online version of this article (doi:10.1186/s40529-014-0049-3) contains supplementary material, which is available to authorized users.

## Background

Processed yam tuber is a traditional Chinese medicine used as a juvenescent substance, with no apparent side effects resulting from long-term use, which is ranked the top levels in Chinese Pharmacopoeia. Yams (*Dioscorea* spp., Dioscoreaceae) are an important tuber crop in Africa, Asia, and Middle and South America and are also a staple food in the Caribbean. Yam dioscorin and its peptic hydrolysates have been reported to exhibit several biological activities *in vitro* and *in vivo* (Lu et al. [2012]), including antioxidant (Hou et al. [2001]; Liu et al. [2006]; Han et al. [2013], [2014a],[b]), antihypertensive (Hsu et al. [2002]; Lin et al. [2006]; Liu et al. [2009a]), immunomodulatory (Liu et al. [2007]; Liu et al. [2009b]), and enzyme activities (Hou et al. [1999a], [b]; Hou et al. [2000]).

The untreated high blood pressure is considered to be the central factor in stroke which accounts approximately 33% deaths (Mark and Davis [2000]). There are several classes of pharmacological agents which have been used in the treatment of hypertension, and one class of antihypertensive drugs known as angiotensin I converting enzyme (ACE) inhibitors is associated with a low rate of adverse side effects and is the preferred class of antihypertensive agents when treating patients with concurrent secondary diseases or cardiovascular diseases (Zaman et al. [2002]). ACE (peptidyldipeptide hydrolase, EC 3.4.15.1) is a dipeptide-liberating Zn-containing exopeptidase, which removes a dipeptide from the C-terminus of angiotensin I to form angiotensin II, a very hypertensive compound. Several ACE inhibitory peptides were isolated from food proteins and exhibited generally to reduce blood pressures of SHRs (Martínez-Maqueda et al. [2012]). Fujita et al. ([2000]) found that the octapeptides of FFGRCVSP (IC_50_ = 0.4 μM) and ERKIKVYL (IC_50_ = 1.2 μM) were potent ACE inhibitors, but none of them were effective in spontaneously hypertensive rats (SHRs) to reduce the blood pressure. These potential ACE inhibitory peptides were further hydrolyzed by the rat’s gastrointestinal proteases and then lose their antihypertensive effects on SHR *in vivo*. We reported that yam dioscorin and its peptic hydrolysates exhibited ACE inhibitory activity (Hsu et al. [2002]) and antihypertensive activity (Lin et al. [2006]) using SHRs as models, however, the active peptides are not really isolated until now. In this study, using angiotensin converting enzyme inhibition as preliminary screenings, two out of twenty-three synthesized peptides from a computer-aided simulation of pepsin hydrolysis of yam dioscorin were selected for further *ex vivo* and *in vivo* experiments and captopril was used for comparisons. It is suggested that KTCGY and KRIHF show vasodilating effects and can reduce SHR’s systolic blood pressure (SBP) which may contribute important roles in yam dioscorin for regulating blood pressure *in vivo*.

## Methods

### Materials

The 23 peptides for ACE inhibitory activity screenings were synthesized by Mission Biotech. (Taipei, Taiwan). Peptides for *ex vivo* and i*n vivo* experiments used in this study (KTCGY, KTCGYKTCGY, TCGYTCGY, and KRIHF) were synthesized by Shanghai Hanhong Chemical Co., Ltd (Shanghai, China) or Shanghai Mocell Biotech Co., Ltd (Shanghai, China). The purity of each peptide was determined to be greater than 97% by performing HPLC chromatography and mass spectroscopy. Captopril was purchased from Calbiochem Co. (CA, USA). *N*-(3-[2-furyl]acryloyl]-Phe-Gly-**G** ly (FAPGG), ACE (I unit, rabbit lung), dimethyl sulfoxide (DMSO) were purchased from Sigma Chemical Co. (St. Louis, MO, USA). Other chemicals and reagents were from Sigma Chemical Co. (St. Louis, MO, USA).

### Computer-aided simulation of pepsin hydrolysis of yam dioscorin

The deduced sequences of dioscorin A (UniProtKB/TrEMBL:Q9M519) and dioscorin B (UniProtKB/TrEMBL:Q9M501) were selected from the website of the ExPASy SIB Bioinformatics Resource Portal (http://www.expasy.org/proteomics) to participate in a computer-aided simulation of pepsin hydrolysis (pH > 2) (http://web.expasy.org/peptide_mass/). Twenty-three peptides were selected for syntheses following the general rules which the peptide contained aromatic or branched-chain aliphatic amino acids closed to the COOH-terminus for ACE inhibitions (Cheung et al. [1980]), including (1) KTCGNGME, (2) PPCSE, (3) CDDRVIRTPLT, (4) KTCGY, (5) PPCTE, (6) RDNGVIF, (7) KRIHF, (8) RRDY, (9) RSVF, (10) PTNF, (11) GISW, (12) MGSF, (13) VSIL, (14) HSPA, (15) DPF, (16) RY, (17) RF, (18) NW, (19) RL, (20) GVI, (21) GSL, (22) SY, and (23) GPA.

### ACE inhibitory assay screenings

The ACE inhibitory activity was measured following the previous reports with some modifications (Hsu et al. [2002]). Each synthesized peptide was dissolved in DMSO to 4 mM as stocks. The 1 ml, 0.5 mM FAPGG (dissolved in 50 mM Tris–HCl buffer, pH 7.5, containing 0.3 M NaCl) was mixed with 12.2 μl peptide solution, and then twenty μl (20 μU) of commercial ACE (stock solution, 1U/ ml) was added. The 0.1% DMSO solution was used instead of sample solution for blank experiments (ΔA_blank_/min). The decreased absorbance at 345 nm (ΔA_sample_/min) was recorded during 90 sec at room temperature. The ACE inhibition (%) was calculated as followed: [1 - (ΔA_sample_/min ÷ ΔA_blank_/min)] × 100.

### Effects of KTCGY, KTCGYKTCGY, TCGYTCGY, and KRIHF on phenylephrine-induced tensions in rat thoracic aortic ring *ex vivo*

Effects of four peptides and captopril on vascular tension were examined following the previous method (Wang et al. [2001]). Male Sprague–Dawley rats were killed by decapitation, and sections of the thoracic aorta between the aortic arch and the diaphragm were excised carefully and placed in a Petri dish with oxygenated Krebs’ buffer (120 mM NaCl, 4.5 mM KCl, 2.5 mM CaCl_2_, 1 mM MgSO_4_, 27 mM NaHCO_3_, 1 mM KH_2_PO_4_, 10 mM glucose, pH 7.4, with 95% O_2_, 5% CO_2_) and gently dissected free of fat and connective tissue. The endothelium was removed by gently rubbing the intimal layer of the vessel with a wire. The isolated aortic rings, 3 to 4 mm in length with or without the endothelium, were fixed isometrically in organ chambers under passive tension (1.8 g) for 60 min. Changes in vascular tension were recorded with a polygraph (Gould, model 2400, Valley View, OH, USA) via a force displacement transducer (Grass FT03, Quincy, MA, USA). After equilibration, the near maximal concentration was induced by PE (0.3 μM). When the rings achieved a stable tension, the acetylcholine (3 μM) was added to the bath to assess endothelial integrity. The relaxing effects of KTCGY, KTCGYKTCGY, TCGYTCGY, KRIHF, and captopril on PE (0.3 μM) pre-contracted endothelium-intact or endothelium-denuded aortic rings were examined. When PE treatments reached a steady plateau (considered as 100%), KTCGY, KTCGYKTCGY, TCGYTCGY, KRIHF, and captopril were then tested at cumulative concentrations (0.1 to 100 μM) for their ability to induce aortic ring relaxations.

### Antihypertensive Effects of KTCGY, KRIHF, and captopril in SHRs

Experimental procedures were reviewed and approved by the Institutional Animal Care and Use Committee of Taipei Medical University (LAC-95-0076, LAC-97-0117, and LAC-100-0038). Male SHRs (18–28 weeks old; from National Laboratory Animal Center, Taipei, Taiwan) were housed individually in steel cages kept at 24°C under a 12-h light–dark cycle, with free access to water and a standard mouse/rat chow (Prolab® RMH2500, 5P14 Diet, PMI Nutrition International Brentwood, MO, USA). SHRs were randomly divided into 4 groups (N = 6 of each group), KTCGY or KRIHF at concentration of 10 mg/kg and 20 mg/kg was orally administered to the SHRs, and the SBP were measured after 0, 2, 4, 6, 8, and 24 h by using an indirect tail-cuff blood pressure meter (BP98-A, Softron, Tokyo, Japan). Distilled water (0.5 ml) was administered to the SHRs in the blank group. The captopril (10 mg/kg) was used as the positive control.

### Statistical analysis

Data were expressed as mean ± S.D. For animal experiments, the differences between the blank and the experimental group at the same time was analyzed using Student’s *t*-test, and the *P*-value of less than 0.05 (*), 0.01 (**), and 0.001 (***) were recognized as different significantly. The statistical analysis was performed using the SigmaPlot software 10.0.

## Results

### ACE inhibitory assay screenings

**Figure 1:**
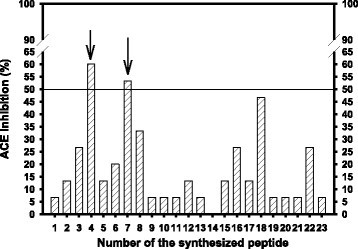
**ACE inhibition by 23 synthesized peptides (40 μM) derived from a computer-aided simulation of pepsin hydrolysis of yam dioscorin.** The 0.1% DMSO solution was used instead of sample solution for blank experiments (ΔA_blank_/min). The decreased absorbance at 345 nm (ΔA_sample_/min) was recorded during 90 sec at room temperature. The ACE inhibition (%) was calculated as followed: [1 - (ΔA_sample_/min ÷ ΔA_blank_/min)] × 100. The synthesized peptides included (1) KTCGNGME, (2) PPCSE, (3) CDDRVIRTPLT, (4) KTCGY, (5) PPCTE, (6) RDNGVIF, (7) KRIHF, (8) RRDY, (9) RSVF, (10) PTNF, (11) GISW, (12) MGSF, (13) VSIL, (14) HSPA, (15) DPF, (16) RY, (17) RF, (18) NW, (19) RL, (20) GVI, (21) GSL, (22) SY, and (23) GPA. Arrow indicated peptide with ACE inhibition over 50%.

The deduced sequences of dioscorin A (UniProtKB/TrEMBL:Q9M519) were selected for computer-aided simulation of pepsin hydrolysis (pH > 2). There were sixty-four peptide fragments and seven amino acid residues (A, L, Q, E, F, Y, and W) were released from dioscorin A in a computer-aided simulation hydrolysis (Additional file 1: Figure S1). The deduced sequences of dioscorin B (UniProtKB/TrEMBL:Q9M501) were selected for simulation of pepsin hydrolysis (pH > 2). There were sixty-five peptide fragments and eight amino acid residues (A, I, L, Q, E, F, Y, and W) were released from dioscorin B in a computer-aided simulation hydrolysis (Additional file 1: Figure S2). Following the general rules which the peptide contained aromatic or branched-chain aliphatic amino acids closed to the COOH-terminus for ACE inhibitions (Cheung et al. [1980]), the twenty-three peptides were selected for syntheses in order to test ACE inhibitory activity. In assay system, the substrate FAPGG was hydrolyzed by ACE to generate FAP and dipeptide GG released, and the absorbance at 345 nm was decreased. From the results of Figure 1, under the same concentration of 40 μM for each peptide, the KTCGY (No.4) and KRIHF (No. 7) showed the first two potent ACE inhibitory peptides among 23 synthesized peptides. The KTCGY and KRIHF with higher than 50% ACE inhibitory activity were selected for further *ex vivo* and *in vivo* antihypertensive experiments. Table 1 showed the information of KTCGY and KRIHF in yam dioscorin.

**Table 1:** **The synthesized peptides derived from computer-aided simulation of pepsin hydrolysis** ^**a**^ **of yam dioscorin** ^**b,c**^ **with potential angiotensin converting enzyme inhibitory activities used in this study**

Synthesized peptide no.	Peptide sequence^a^	Protein sources^b,c^	Molecular mass (Average mass, Da)
4	KTCGY	dioscorin B (52–56)	570.66
7	KRIHF	dioscorin A (118–122)	699.85
		dioscorin B (118–122)	

^a)^PeptideMass tool in Proteomics (http://web.expasy.org/peptide_mass/) of ExPASy: SIB Bioinformatics Resource Portal (http://www.expasy.org) website.

^b)^Dioscorin A from UniProtKB/TrEMBL:Q9M519;

^c)^Dioscorin B from UniProtKB/TrEMBL:Q9M501.

### Relaxations in rat thoracic aortic ring with or without endothelium *ex vivo*

**Figure 2:**
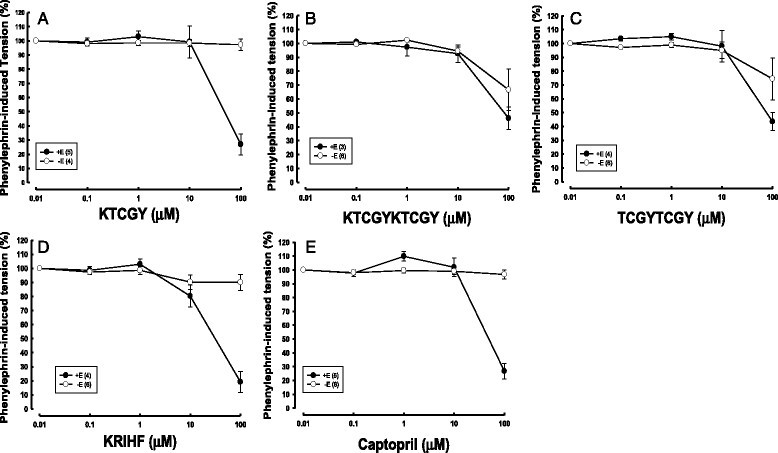
**Vasorelaxing activity of endothelium-intact and endothelium-denuded SD rat aortic rings in phenylephrine-induced tensions. (A)** KTCGY, **(B)** KTCGYKTCGY, **(C)** TCGYTCGY, **(D)** KRIHF, and **(E)** captopril on Data were expressed as mean ± S.E.

Except from two penta-peptides of KTCGY and KRIHF, KTCGYKTCGY (two-repeated KTCGY) and TCGYTCGY (two-repeated TCGY) were also synthesized for *ex vivo* aortic ring vasodilating experiments and captopril was used for comparisons. It was found that KTCGY, KRIHF, and captopril showed endothelium-dependent, but not endothelium-denuded, aortic ring relaxations in PE-induced tensions (Figure 2A, D, and E). When 100 μM of KTCGY, KRIHF, or captopril was added, the PE-induced aortic ring tension was markedly reduced from 100% to (26.97 ± 7.48)%, (19.19 ± 7.63)% or (26.81 ± 5.54)%, respectively. Only KRIHF at 10 μM could reduce PE-induced aortic ring tensions from 100% to (80.26 ± 7.82)%. However, KTCGYKTCGY and TCGYTCGY showed endothelium-independent aortic ring relaxations in PE-induced tensions (Figure 2B and C). When 100 μM of KTCGYKTCGY or TCGYTCGY was added, the PE-induced endothelium-intact aortic ring tension was markedly reduced from 100% to (46.05 ± 8.02)% or (43.52 ± 6.42)%, respectively; under the same 100 μM concentration, KTCGYKTCGY or TCGYTCGY reduced the PE-induced endothelium-denuded aortic ring tension from 100% to (66.59 ± 14.84)% or (74.44 ± 15.13)%, respectively.

### Antihypertensive activity *in vivo*

**Figure 3:**
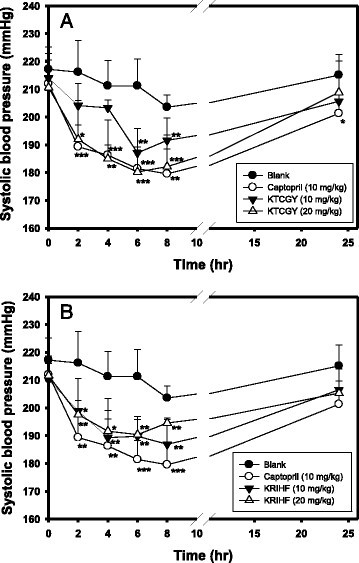
**Antihypertensive activity of (A) KTCGY and (B) KRIHF on systolic blood pressure of spontaneously hypertensive rats (SHRs).** KTCGY or KRIHF at concentration of 10 mg/kg and 20 mg/kg was orally administered to the SHRs (6 rats/group), and the SBP were measured after 0, 2, 4, 6, 8, and 24 h by using an indirect tail-cuff blood pressure meter (BP98-A, Softron, Tokyo, Japan). Distilled water (0.5 ml) was administered to the SHRs in the blank group. The captopril (10 mg/kg) was used as the positive control. Data were expressed as mean ± S.D. For animal experiments, the differences between the blank and the experimental group at the same time was analyzed using Student’s *t*-test, and the *P*-value of less than 0.05 (*), 0.01 (**), and 0.001 (***) were recognized as different significantly.

The KTCGY or KRIHF (10 or 20 mg/kg) was orally administered once and the SBP was measured at 2-, 4-, 6-, 8-, and 24-h, and the captopril (10 mg/kg) was used for comparisons. Figure 3A shows the SBP changes of SHR after KTCGY oral administration during 24 h. It was found that KTCGY at 10 or 20 mg/kg showed dose-dependently antihypertensive activity by lowing SBP and showed significantly different compared to the blank at 6-h (*P* < 0.01) and 8-h (*P* < 0.01) for 10 mg/kg and at 2-h (*P* < 0.05), 4-h (*P* < 0.001), 6-h (*P* < 0.001), 8-h (*P* < 0.001) for 20 mg/kg. The average reduced SBP for 10 mg/kg at 6-h and 8-h was 24.17 and 11.93 mmHg, respectively; the average reduced SBP for 20 mg/kg at 2-h, 4-h, 6-h, and 8-h was 24.20, 26.24, 31.03, and 21.40 mmHg, respectively. The positive control of captopril (10 mg/kg) showed antihypertensive activity by lowing SBP and showed significantly different compared to the blank at 2-h (*P* < 0.001), 4-h (*P* < 0.01), 6-h (*P* < 0.001), 8-h (*P* < 0.01), and 24-h (*P* < 0.05). The average reduced SBP for captopril (10 mg/kg) at 2-h, 4-h, 6-h, 8-h, and 24-h was 26.85, 24.97, 29.82, 24, and 13.80 mmHg, respectively. It was noted that KTCGY at 20 mg/kg showed similar antihypertensive activity to captopril at 10 mg/kg. Figure 3B shows the SBP changes of SHR after KRIHF oral administration during 24 h. It was found that KRIHF at 10 or 20 mg/kg showed dose-independently antihypertensive activity by lowing SBP and showed significantly different compared to the blank at 2-h (*P* < 0.05), 4-h (*P* < 0.01), 6-h (*P* < 0.01) and 8-h (*P* < 0.01) for 10 mg/kg and at 2-h (*P* < 0.01), 4-h (*P* < 0.05), 6-h (*P* < 0.01), 8-h (*P* < 0.01) for 20 mg/kg. The KRIHF at dose of 10 and 20 mg/kg showed similar antihypertensive activities toward SHRs. The average reduced SBP for 10 mg/kg at 2-h, 4-h, 6-h, and 8-h was 17.30, 21.95, 21.47, and 16.65 mmHg, respectively; the average reduced SBP for 20 mg/kg at 2-h, 4-h, 6-h, and 8-h was 18.57, 19.64, 20.87, and 8.99 mmHg, respectively.

## Discussion

The present *ex vivo* and *in vivo* data showed KTCGY and KRIHF with vasodilating effects against PE-induced endothelium-intact aortic tensions and lowering SHR’s SBP which were derived from a computer-aided simulation of pepsin hydrolysis of the yam tuber dioscorin. The penta-peptide of KTCGY was positioned at amino acid residue 52 to residue 56 of dioscorin B protein (UniProtKB/TrEMBL:Q9M501, Fig. S2), and the penta-peptide of KRIHF was positioned at amino acid residue 118 to residue 122 of dioscorin A protein (UniProtKB/TrEMBL:Q9M519, Fig. S1) and dioscorin B protein (UniProtKB/TrEMBL:Q9M501, Fig. S2). Conlan et al. ([1995]) first reported two classes of cDNA clones encoding major yam tuber storage proteins from *Dioscorea cayenensis* and named as dioscorin. We demonstrated that dioscorins from six cultivars of three *Dioscorea* species accounted over 90% of water-soluble proteins by immune stains (Hou et al. [2000]). The processed yam tuber is a traditional Chinese medicine used as a juvenescent substance, with no apparent side effects after long-term uses; the tuber is involved in the top levels in a famous Chinese pharmacopoeia. It was recently reviewed that yam dioscorin and its peptic hydrolysates exhibited several biological activities *in vitro* and *in vivo* (Lu et al. [2012]). It seems that yam or its major protein, dioscorin, may be potentials in developments of several aspects of functional food and needed further investigations.

From the present results of ACE inhibitory activity screenings (Figure 1), the orders of top 5 among 23 synthetic peptides were KTCGY (No.4) > KRIHF (No. 7) > NW (No. 18) > RRDY (No. 8) > RY (No. 16) ≅ SY (No. 22) ≅ CDDRVIRTPLT (No. 3). These potent ACE inhibitory peptides were promised to the general rules which peptides contained aromatic or branched-chain aliphatic amino acids closed to the COOH-terminus for ACE inhibitions (Cheung et al. [1980]). The dioscorin peptic hydrolysates were reported to exhibit ACE inhibitory activity (Hsu et al. [2002]) and antihypertensive activity (Lin et al. [2006]) using SHR as models. Therefore, the synthetic KTCGY and KRIHF were used for *ex vivo* and *in vivo* experiments. KTCGY and KRIHF showed endothelium-dependent aortic ring relaxations in PE-induced tensions (Figure 2A, D) which the vasodilating effects were similar to ACE inhibitor, captopril (Figure 2E). The captopril is designed to inhibit ACE, one of important enzymes in renin-angiotensin system, to achieve antihypertension (Rubin et al. [1978]). It was reported that captopril also showed endothelium-dependent rabbit aortic ring relaxations in PE-induced tensions, and the possible action mechanism might be from its superoxide radical scavenging activity of its structural sulfhydryl group (Mittra and Singh [1998]). Hernández-Ledesma et al. ([2005]) reported that individual amino acids exhibited different oxygen radical absorbance capacity (ORAC) activities, such as Trp [W, 4.649 μmole trolox equivalents (TE)/μmole], Tyr (Y, 1.574 μmole TE/μmole), Met (M, 1.126 μmole TE/μmole), Cys (0.1492 μmole TE/μmole), His (H, 0.073 μmole TE/μmole), and Phe (F, 0.0025 μmole TE/μmole), which a Trp-indole group, Tyr-phenolic group, His-imidazole group, and Cys-sulfhydryl group served as hydrogen donors to contribute ORAC activity. It was reported that the ovokinin (2–7) (a hexa-peptide, RADHPF) isolated from chymotryptic ovalbumin hydrolysates exhibited dose-dependently nitric oxide-stimulated vaso-relaxations in endothelium-intact PE-induced tensions of SHR’s mesenteric artery, however, ovokinin (2–7) showed less effect on ACE inhibition (IC_50_ higher than 1 mM) (Matoba et al. [1999]). Therefore, we propose that vaso-relaxing peptides of KTCGY and KRIHF in endothelium-intact aortic rings may involve radical scavenging and nitric oxide stimulating activities and need further investigations.

The KTCGYKTCGY and TCGYTCGY showed endothelium-intact and endothelium-denuded vaso-relaxing activities in PE-induced tensions which were different from the action mode of KTCGY of above-mentioned. The ovokinin (an octa-peptide, FRADHPFL) isolated from peptic hydrolysates of egg ovalbumin showed bradykinin B1 receptor antagonist and exhibited endothelium-independent canine mesenteric artery relaxations in prostaglandin F2α-induced tensions (Fujita et al. [1995]). Recently, a series of experiments by Matsui team demonstrated that WH di-peptide showed endothelium-independent SD rat aortic ring relaxations in KCl-induced contractions by suppressing Ca^2+^ influx (Tanaka et al. [2008]), which the inhibition of extracellular calcium entry into vascular smooth muscle cells by blocking dihydropyridine-like L-type Ca^2+^ channels (Wang et al. [2010]), and the inhibition against voltage-dependent L-type Ca^2+^ channels phosphorylation (Kobayashi et al. [2012]). The HRW tri-peptide also showed endothelium-independent vaso-relaxing activities in PE-induced tensions by inhibiting extracellular Ca^2+^ influx (Tanaka et al. [2009]). Therefore, it is proposed that KTCGYKTCGY and TCGYTCGY with endothelium-independent vaso-relaxing activities in PE-induced tensions might involve Ca^2+^ influx and Ca^2+^ channels and needed investigations further.

From the present results of *in vivo* SHR oral administration, both penta-peptides of KTCGY and KRIHF at doses of 10 and 20 mg/kg exhibited antihypertensive activities by lowering SBP, but not diastolic blood pressure (data not shown), among which KTCGY of 20 mg/kg exhibited the similar lowering SBP profile to captopril of 10 mg/kg after a single oral administration. However, the vaso-relaxing peptide with antihypertensive activity is not necessary for potent ACE inhibition. Therefore, it might loss some antihypertensive peptides from ACE inhibitory screenings in the present study. The RF di-peptide (Kagebayashi et al. [2012]) and IHRF tetra-peptide (Kontani et al. [2014]) isolated from rice glutelin with lower ACE inhibition was reported to exhibit cholecystokinin-dependent vaso-relaxing and antihypertensive activities in SHR, which the RF di-peptide was the same as No. 17 synthesized peptide in Figure 1 of less ACE inhibitory activity in the present study from computer-aided simulation of pepsin hydrolysis of yam dioscorin A (residues of 134–135 and 158–159) and yam dioscorin B (residues of 158–159). However, these results provide evidences to support yam dioscorin after ingestion for blood pressure regulations.

## Conclusions

The KTCGY and KRIHF show vasodilating effects and can reduce SHR’s systolic blood pressure which may contribute important roles in yam dioscorin for regulating blood pressure *in vivo* and will be beneficial for anti-hypertension in functional food preparations.

## Additional file

## Electronic supplementary material

Additional file 1: Figure S1.: The computer-aided simulation of pepsin hydrolysis of yam dioscorin A (Q9M519). **Figure S2.** The computer-aided simulation of pepsin hydrolysis of yam dioscorin B (Q9M501). (DOCX 905 KB)

Below are the links to the authors’ original submitted files for images.

Authors’ original file for figure 1

Authors’ original file for figure 2

Authors’ original file for figure 3
