# Supplementary material for: Vasorelaxing and antihypertensive activities of synthesized peptides derived from computer-aided simulation of pepsin hydrolysis of yam dioscorin
Source: Bot Stud. 2014 Jun 7;55:49. doi: 10.1186/s40529-014-0049-3 (PMC5432742; doi:10.1186/s40529-014-0049-3)
Supplement: Supplementary file 1 — Additional file 1: Figure S1.: The computer-aided simulation of pepsin hydrolysis of yam dioscorin A (Q9M519). Figure S2. The computer-aided simulation of pepsin hydrolysis of yam dioscorin B (Q9M501). (DOCX 905 KB) [file 40529_2014_49_MOESM1_ESM.docx]

Figure S1. The computer-aided simulation of pepsin hydrolysis of yam dioscorin A (Q9M519).

Figure S2. The computer-aided simulation of pepsin hydrolysis of yam dioscorin B (Q9M501).
