# Supplementary figures and images for: Vasorelaxing and antihypertensive activities of synthesized peptides derived from computer-aided simulation of pepsin hydrolysis of yam dioscorin
Source: Bot Stud. 2014 Jun 7;55:49. doi: 10.1186/s40529-014-0049-3 (PMC5432742; doi:10.1186/s40529-014-0049-3)

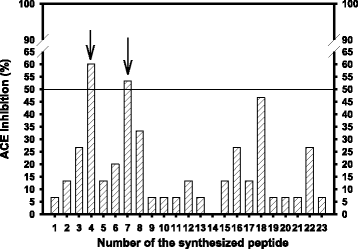

Supplement: Supplementary file 2 — Authors’ original file for figure 1 [file 40529_2014_49_MOESM2_ESM.gif]

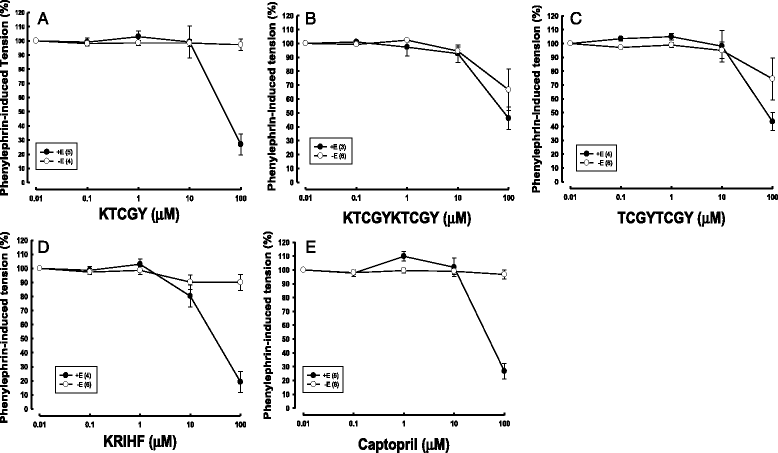

Supplement: Supplementary file 3 — Authors’ original file for figure 2 [file 40529_2014_49_MOESM3_ESM.gif]

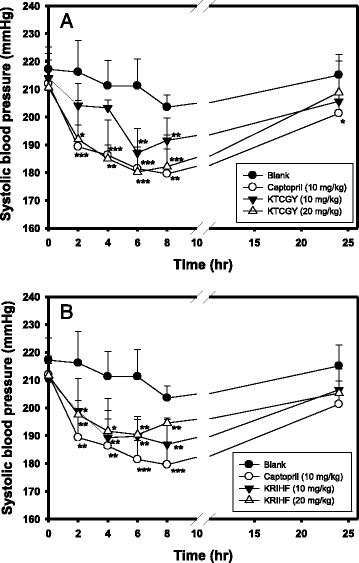

Supplement: Supplementary file 4 — Authors’ original file for figure 3 [file 40529_2014_49_MOESM4_ESM.gif]
